# Application of Ventana immunocytochemical analysis on ThinPrep cytology slides for detection of ALK rearrangement in patients with advanced non–small-cell lung cancer

**DOI:** 10.1186/s12885-018-5184-x

**Published:** 2018-12-20

**Authors:** Hui Qin Guo, Jia Jia, Lin Lin Zhao, Huan Zhao, Cong Wang, Yue Sun, Jian Ming Ying, Lei Guo, Jian Cao, Zhi Hui Zhang

**Affiliations:** Department of Pathology, National Cancer Center/National Clinical Research Center for Cancer/Cancer Hospital, Chinese Academy of Medical Sciences and Peking Union Medical College, Chaoyang District, Panjiayuan 17 in the South, Beijing, 100021 China

**Keywords:** ALK rearrangement, Non–small-cell lung cancer, ThinPrep slides, Semiquantified interpretation system, Binary scoring algorithm

## Abstract

**Background:**

Ventana ALK (D5F3) screening of anaplastic lymphoma kinase (ALK) gene rearrangement in tissue specimens has been approved by US FDA (Food and Drug Administration) to select treatment for non–small-cell lung carcinoma (NSCLC). However, tumor tissues are often not readily obtainable, and cytology specimens and may be the only tumor material available for diagnosis and molecular marker analysis. In this study, we evaluated the feasibility of ALK immunocytochemistry (ICC) on ThinPrep slides and determined a suitable scoring system for interpretation of the results.

**Methods:**

One hundred twenty-one fine-needle aspirate (FNA) specimens from metastatic lesions of NSCLC were analyzed. ALK rearrangement was detected on ThinPrep cytology slides using the Ventana immunocytochemistry ALK-D5F3 system, which adopts two scoring systems for interpretation of the ICC results. The results were subsequently confirmed by reverse transcription polymerase chain reaction (RT-PCR) analysis and fluorescence in situ hybridization (FISH).

**Results:**

Among the 121 ICC specimens, 16 that were considered ALK-positive by either scoring system were referred for PCR analysis. Among the ALK ICC-negative cases, 33 had correlated FISH ALK results. A total of 49 specimens that exhibited either a positive or negative ICC result with a correlated ALK status were analyzed statistically. ICC results showed a high concordance rate with the results of PCR/FISH analysis. The sensitivity and specificity of ALK ICC by the binary scoring algorithm were 68.75 and 96.97%, respectively. These values increased to 93.75 and 96.97%, respectively, when interpreted by the semiquantified interpretation system.

**Conclusions:**

ALK ICC analysis on ThinPrep slides is a reliable ALK testing method, and the semiquantified interpretation system on cytology specimens is recommended rather than the binary scoring algorithm on tissue specimens.

## Background

In the past decade, personalized medicine directed at specific molecular targets in tumors has helped improve the survival of patients with non–small-cell lung cancer (NSCLC) [1, 2]. For the treatment of patients with NSCLC harboring anaplastic lymphoma kinase (ALK) gene fusions, crizotinib, ceritinib, and alectinib have all been approved*.* ALK rearrangements have been reported in 3%-7% of lung tumors [3–5]. In clinical practice, 3 methods of detecting ALK rearrangements are generally used: fluorescence in situ hybridization (FISH), reverse-transcription polymerase chain reaction (RT-PCR) assays, and immunohistochemistry (IHC) analysis. Of these, IHC is easily practicable and less costly, and has been approved by the US FDA as a screening assay for detecting ALK rearrangements [6–9]. Currently, most studies of IHC have used tissue sections and only a few have focused on the feasibility of IHC using cytology specimens [10].

As lung cancer is only diagnosed at an advanced stage in many patients, there is often no opportunity for surgery. Consequently, tissue sections may not be readily obtainable and cytology specimens are sometimes the only tumor material available for diagnosis and molecular marker analysis [11–13]. While formalin-fixed, paraffin-embedded cell blocks using the same processing methods as for tissue sections are ideal for IHC analysis, not all cytology materials are sufficient for cell block processing [14]. In such cases, direct smears or liquid-based slides such as ThinPrep, which are used for cytological diagnosis, may be the only available material for molecular marker analysis. Although ALK ICC analysis using direct cytology smears has been described previously [10, 15], to our knowledge, there are no published studies of the application of ALK ICC analysis on ThinPrep (liquid-based) slides.

In the present study, we performed ALK ICC analysis on ThinPrep slides, with the results interpreted by 2 separate scoring systems [7, 16]. To evaluate the accuracy of ALK ICC analysis on ThinPrep slides, the results with both scoring systems were correlated with ALK PCR/FISH results. We also determined the most suitable of the 2 scoring systems for ALK ICC analysis on ThinPrep slides.

## Methods

### Patient selection

A consecutive series of NSCLC patients evaluated in the Department of Pathology, National Cancer Center/Cancer Hospital, Chinese Academy of Medical Sciences, Beijing between November 2013 and October 2016 comprised the study cohort. Specimens from the patients were selected for analysis on the following basis: (1) fine-needle aspirate (FNA) specimens were able to be obtained from metastatic lesions; (2) at least 15 mL of PreservCyt solution (Hologic Inc., Marlborough, MA, USA) was left in the specimen bottles; and (3) more than 100 tumor cells were present in Papanicolaou-stained ThinPrep slides (Hologic Inc, Marlborough, MA, USA) [17]. The liquid-based materials were stored at 4°C and submitted for ICC and molecular testing within 3 months.

The study protocol was reviewed and approved by the ethics committee of the National Cancer Center/Cancer Hospital, Chinese Academy of Medical Sciences. All patients provided informed consent to participate in the study.

### Immunocytochemistry (ICC) analysis

Liquid-based specimens were required to be at room temperature for 20 minutes before preparation of the ThinPrep slides. Automated ICC for ALK expression was performed on ThinPrep slides (each sample had 2 slides) using the highly sensitive anti-ALK (D5F3) rabbit monoclonal primary antibody and a corresponding negative control (Ventana Medical Systems Inc., Tucson, AZ, USA). The entire staining procedure was performed according to the manufacturer’s instructions on a BenchMark XT slide stainer (Ventana Medical Systems, Inc.).

**Fig. 1:**
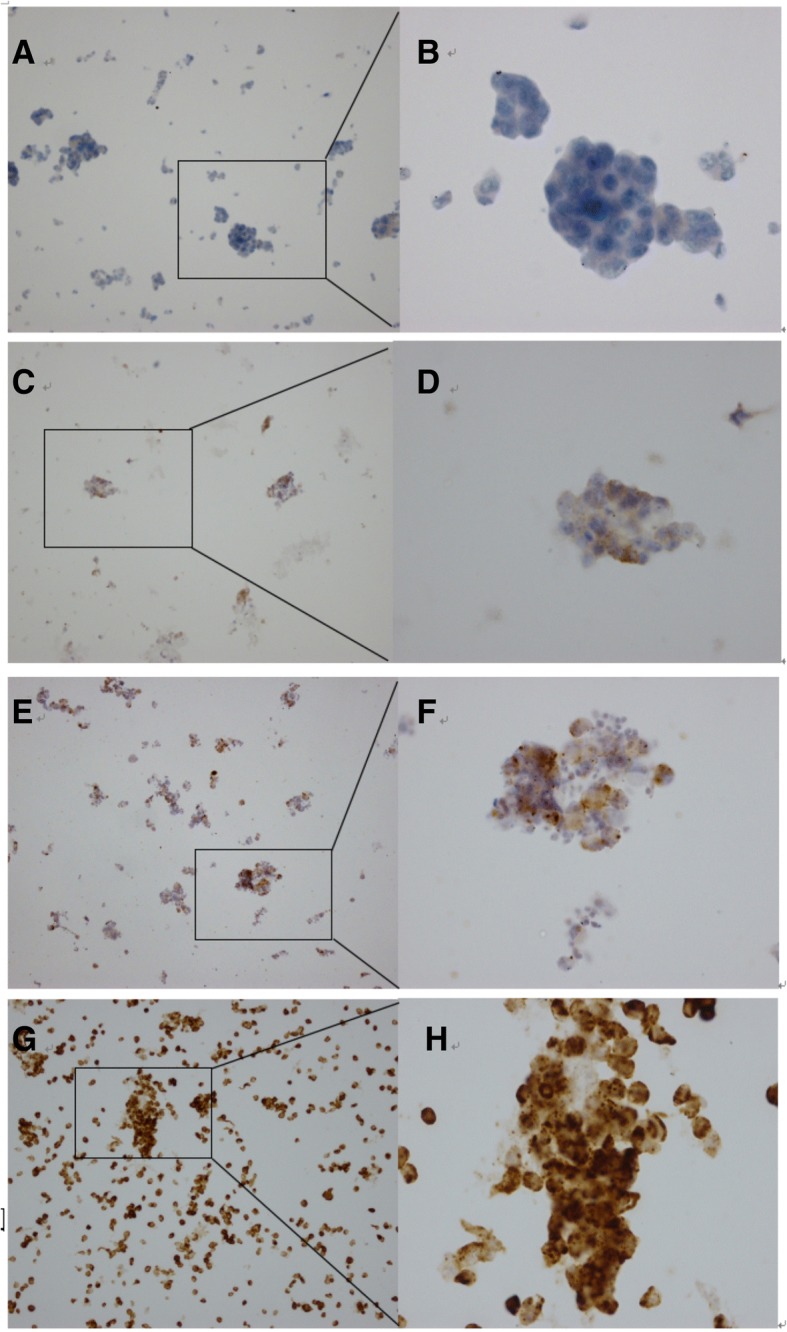
Semiquantified interpretation system compared with the binary system. **a** ALK staining patterns zero (no stain, 200´ magnification), **b** zero ( 400´). **c** 1+ (uncertain weak stain seen at 200´ magnification), **d** 1+ (weak cytoplasmic stain seen at 400´ magnification). **e** 2+ (moderate cytoplasmic stain seen at 200´ magnification), **f** 2+ (400´). **g** 3+ (strong cytoplasmic stain seen at 40´ magnification), **h** 3+ (400´). Scores of 2+ and 3+ are considered ALK positive, while scores of zero and 1+ are considered negative by the binary system

For interpretation of the Ventana ALK (D5F3) ICC results, 2 scoring systems were adopted. The first of these, the binary scoring algorithm, which is recommended by the manufacturer, provides positive or negative results based on the granular cytoplasmic staining intensity. Any percentage of tumor cells presenting with strong granular cytoplasmic staining denotes positive ALK expression. The second system, the semiquantified interpretation system, rates ALK staining patterns as zero (no stain), 1+ (weak cytoplasmic stain seen at 400× magnification), 2+ (moderate cytoplasmic stain seen at 100× to 200× magnification), or 3+ (strong cytoplasmic stain seen at 20× to 40× magnification). Scores of 2+ and 3+ are considered ALK-positive, while scores of zero and 1+ are considered negative [10] (Fig. 1).

The staining intensity was scored according to both standards, and the results were reviewed independently by 2 experienced cytopathologists. If there was any discordance between them, the results were re-evaluated. Positive results determined by either scoring system were referred for RT-PCR analysis to confirm the ALK status.

### Reverse-transcription polymerase chain reaction (RT-PCR) analysis

For RT-PCR analysis, cells in PreservCyt solution were first centrifuged, and the RNA was then extracted by a commercial RNA kit [AmoyDx® Tissue RNA Kit (Spin Column), Amoy Diagnostics Co., China]. The RNA quality control assessed by ratio of OD260 to OD280 ranged from 1.9 to 2.0, and the extracted RNA suggested immediate detection. The detection of EML4-ALK*,* including reverse transcriptase and PCR amplification, was performed by RT-PCR according to the manufacturer’s instructions using an AmoyDx® EML4-ALK Fusion Gene Detection Kit. If any EML4-ALK reaction has obvious sample FAM signal amplification curve and Ct <30, then the sample was positive for fusion gene. If all the sample EML4-ALK reaction holes have no FAM signal expansion, growth curve, or Ct = 30, the sample for detection of fusion gene is negative.

### Fluorescence *In Situ* Hybridization (FISH) analysis

FISH was performed on tissue FFPE samples, using the Vysis LSI ALK Dual color Break Apart Probe, according to the manufacturer’s instructions. The LSI ALK 5′ probe (spectrum green) and the LSI ALK 3′ probe (spectrum orange) were applied, hybridized, and assessed along with standard controls according to our standard laboratory procedure and validation studies. A positive cell was defined as one in which the nucleus had split signals (three or more signal diameters apart) or a single orange signal (deleted green signal). A positive sample was considered when if >25 positive cells out of 50 were positive or if there was an average percentage of positive cells of ≥15% in 100 tumour cells. The results were reviewed independently by two experienced pathologists, and any discordance was reevaluated.

### Statistical analysis

The ALK ICC results obtained with the binary scoring algorithm and the semiquantified interpretation system were measured with Cohen’s *k* coefficient. Differences between the ICC interpretations with the 2 scoring systems were analyzed with a χ^2^ test. All statistical analyses were performed with SPSS® 19.0 (SPSS Inc, Chicago, IL, USA). A *P* value <0.05 was considered statistically significant.

## Results

### Patient characteristics

A total of 121 FNA specimens were obtained from 121 NSCLC patients with near or distant metastases, including specimens from supraclavicular lymph nodes (*n* = 106), mediastinal lymph nodes (*n* = 10), axillary lymph nodes (*n* = 2), the chest wall (*n* = 2), and liver (*n* = 1). The 121 patients included 75 males and 46 females, and their median age was 58 years. Histological diagnoses included metastatic adenocarcinoma in 83.47% of the patients (101/121), squamous cell carcinoma in 9.92% (12/121), and non–small-cell-lung cancer not otherwise specified in 6.61% (8/121) (Table 1).

**Table 1:** Patient characteristics

Characteristics	*n*
Mean age, years	58
Sex:	
Male	75
Female	46
Histopathological subtypes:	
Squamous carcinomas	12
Adenocarcinoma	101
Non–small-cell carcinoma NOS	8
Location:	
Supraclavicular lymph nodes	106
Mediastinal lymph nodes	10
Axillary lymph nodes	2
Chest wall	2
Liver	1

*NOS* not otherwise specified

### Detection of ALK rearrangements

Among the 121 specimens analyzed, 16 that were considered ALK-positive by ICC analysis (with either interpretation system) were then referred for PCR analysis, which confirmed an ALK-positive result in 15 cases. The other 105 cytology specimens that were negative for ALK rearrangement by ICC analysis did not undergo any further molecular analysis of the FNA cytology specimens. However, when the medical records of these 105 patients were reviewed, 33 had results for ALK FISH analysis of formalin-fixed tissue specimens. Among the corresponding 33 ALK ICC-negative specimens, 32 were confirmed as negative by ALK FISH analysis of formalin-fixed tissue, but 1 specimen was found to be ALK-positive.

Altogether, a total of 49 specimens that exhibited either a positive or negative ALK rearrangement status in cytology or tissue specimens were included in the statistical analysis to evaluate the feasibility of ALK ICC testing on ThinPrep slides.

### Diagnostic ability of the 2 scoring systems for interpreting ALK ICC results

The binary scoring algorithm for interpreting ALK ICC results detected the expression of ALK protein in 12 of the 121 specimens. Of these, 11 were confirmed as positive for ALK rearrangements by PCR analysis, but 1 proved negative. Five specimens interpreted as ALK-negative by the binary scoring algorithm showed ALK rearrangement on ALK PCR analysis (Table 2). One specimen interpreted as ALK-negative by this interpretation system was ALK-positive on FISH analysis of a tissue specimen (Table 3). Thus, the sensitivity of ALK ICC analysis using the binary scoring algorithm was 68.75% (11/16) and the specificity was 96.97% (32/33). The positive predictive value (PPV) of the test was 91.67% (11/12), and the negative predictive value (NPV) was 86.49% (32/37).

**Table 2:** Comparison of the results obtained with *ALK* ICC analysis using the binary scoring algorithm and *ALK* PCR/FISH analysis

*ALK* ICC result	*ALK* PCR/FISH result		Total
	Positive	Negative	
Positive	11	1	12
Negative	5	32	37
Total	16	33	49

Abbreviations: *ALK*, anaplastic lymphoma kinase, *FISH* fluorescence *in situ* hybridization*, ICC* immunocytochemistry, *IHC* immunohistochemistry, *RT-PCR* reverse-transcription polymerase chain reaction

**Table 3:** Comparison of the results obtained with ALK ICC analysis using the semiquantified interpretation system and ALK PCR/FISH analysis

ALK ICC result	ALK PCR/FISH result		Total
	Positive	Negative	
Positive	15	1	16
Negative	1	32	33
Total	16	33	49

Abbreviations: *ALK* anaplastic lymphoma kinase, *FISH* fluorescence *in situ* hybridization, *ICC* immunocytochemistry, *IHC* immunohistochemistry, *RT-PCR* reverse-transcription polymerase chain reaction

With the semiquantified interpretation system, ALK ICC analysis detected ALK protein expression in 16 of the 121 specimens. Of these, 15 were confirmed as positive for ALK rearrangement by ALK PCR analysis, but 1 proved negative. One specimen interpreted as ALK-negative by the semiquantified interpretation system was ALK-positive on FISH analysis of a tissue specimen (Table 3). Thus, the sensitivity, specificity, PPV, and NPV of the semiquantified interpretation system were 93.75% (15/16), 96.97% (32/33), 93.75% (15/16), and 96.97% (32/33), respectively.

When analyzed statistically, the results obtained with the binary scoring algorithm were not significantly different from those obtained with the semiquantified interpretation system (*P* = 0.125; κ = 0.802), and the sensitivity and specificity of the 2 methods were similar (*P* = 0.169 and *P* = 1.000, respectively).

### Comparison of the 2 scoring systems for interpreting ALK ICC results

**Fig. 2:**
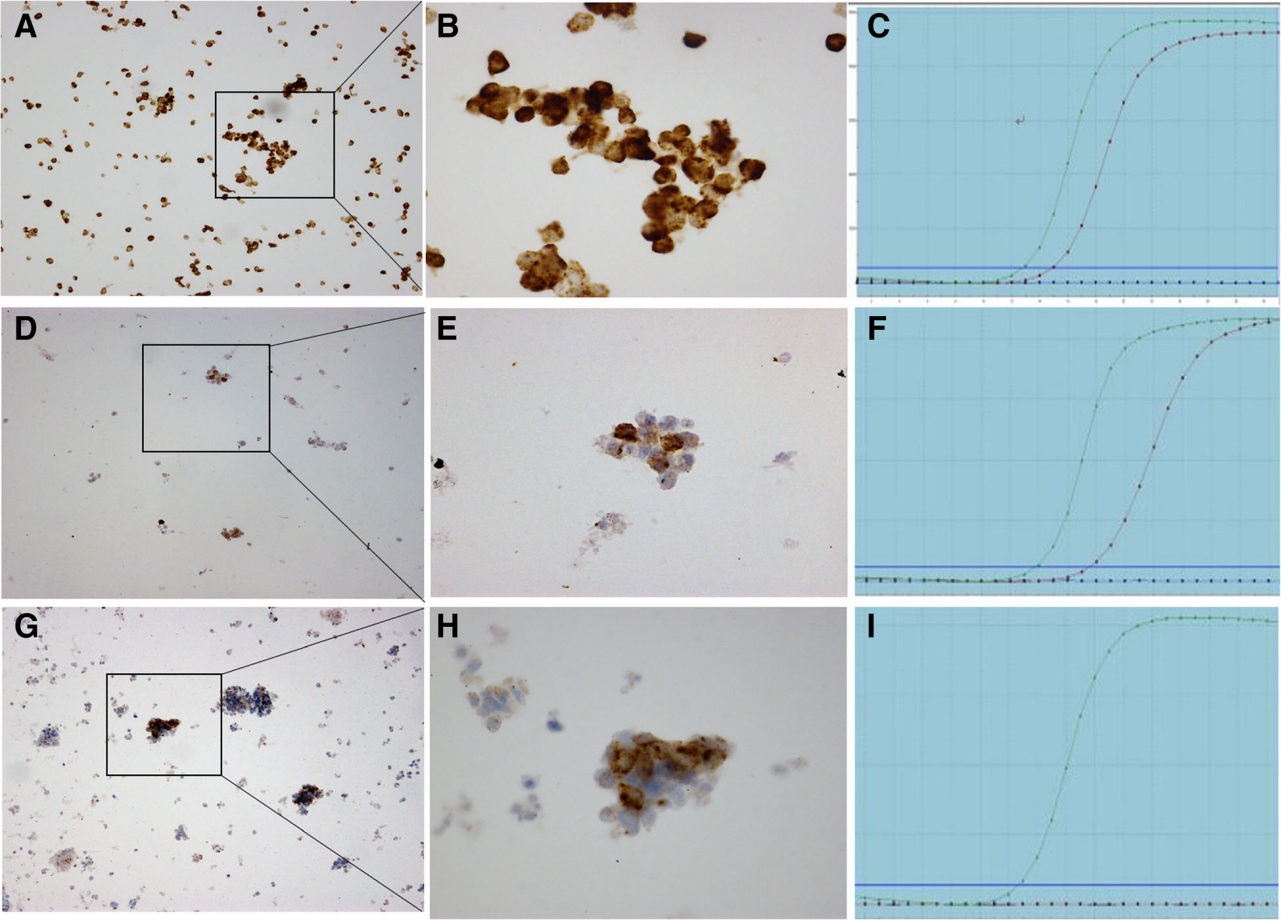
Typical ALK ICC and PCR patterns. **a**, **b**, **c**: Specimens with a positive (score 3+) ALK ICC and *ALK* PCR result. Strong immunostaining was evident in 100% of tumor cells (**a** original magnification, 100×; **b** original magnification, 400×). **d**, **e**, **f**: Specimens with a positive (score 2+) ALK ICC and ALK PCR result. Moderate immunostaining was evident in 5% of tumor cells and faint immunostaining in 95% (**d** original magnification, 100×; **e** original magnification, 400×). **g**, **h**, **i**: Specimens with a false-positive (score 2+) ALK ICC result. Strong immunostaining was evident in 5% of tumor cells and faint immunostaining in 50% (**g**: original magnification, 100×; **h**: original magnification, 400×)

Table 4 shows a comparison of the results obtained with the 2 scoring systems. Among 121 specimens analyzed, there were concordant results with the 2 scoring systems in 117 cases, including 12 positive and 105 negative positive cases; 75% (9/12) of ALK-positive specimens interpreted by the binary scoring algorithm were scored as 3+ by the semiquantified interpretation system, and all were confirmed as positive for ALK rearrangement by PCR analysis (Fig. 2a, b, and, c). One case that was ALK-negative by PCR analysis was interpreted as positive (score 2+) by the 2 interpretation systems (Fig. 2g, h, and i).

**Table 4:** Correlation of the two scoring systems for ALK ICC analysis

Binary scoring algorithm interpretation	Semiquantified interpretation score				Total
	Negative	Positive			
	0	1+	2+	3+	
Positive	0	0	3	9	12
Negative	95	10	4	0	109
Total	95	10	7	9	121

Abbreviations: *ALK* anaplastic lymphoma kinase, *ICC* immunocytochemistry

There were 4 discrepant results between the semiquantified interpretation system and the binary scoring algorithm: all were scored as 2+ by the semiquantified interpretation system but negative by the binary scoring algorithm. The ICC staining patterns for these 4 specimens showed intratumoral heterogeneity. A small proportion of the tumor cells (5%-30%) showed moderate staining intensity (score 2+) but the others (70%-95%) showed faint staining intensity (score 1+). Unexpectedly, all were positive for ALK rearrangement on PCR analysis (Fig. 2d, e, and f)

## Discussion

The Ventana ALK (D5F3) CDx assay (Ventana Medical Systems Inc., Tucson, AZ, USA) has been approved for screening of ALK rearrangement in China and the US since 2012 and 2015, respectively. This standardized IHC assay has been shown to have between 93% and 100% agreement with the results of FISH analysis when used for screening of ALK rearrangement in clinical practice [6, 7].

Cytology specimens play an important role in the diagnosis of lung cancer, especially for inoperable patients, and may be the only tumor material available for diagnosis and molecular marker analysis in up to 44% of cases [11, 12]. The fixative type is an important consideration when using the Ventana ALK (D5F3) CDx assay on cytology specimens, and the one approved by the FDA is formalin-fixed, paraffin-embedded (FFPE) tissue. Studies have shown that alcohol or methanol fixation may cause the loss or a decrease of antigen immunogenicity [16, 18].

Several studies have performed ALK ICC testing on conventional cytology smears and proved the feasibility of the method. In a study by Tanaka et al.[14], 18 cytology smears obtained by bronchoscopic brushing were examined, and the results of ALK ICC analysis on Papanicolau-stained and alcohol-fixed slides were compared with IHC analysis of FFPE tissues. The ICC and IHC results showed a high concordance rate, and the sensitivity and specificity of the ICC test were 85.7% and 100%, respectively [14]. In another study of ALK ICC analysis on direct cytology smears [10], the cytology specimens were fixed in Delaunay solution. The ALK ICC results were correlated with ALK FISH results on the cytology smears, and the sensitivity and specificity of the ICC test were 93.3% and 96.0%, respectively [10].

Since the approval of ThinPrep slides by the US FDA for processing non-gynecological material in 1991, a growing number of laboratories are processing FNA, bronchial brushing, and effusion specimens with this method [19, 20]. In our laboratory, ThinPrep specimens are the only available cytology material for bronchial brushing and imaging-guided FNA specimens. However, there are no published data on the performance of ALK ICC analysis on ThinPrep slides. ThinPrep slides are methanol pre-fixed and alcohol-fixed cytology specimens, which are different to conventional cytology smears. Consequently, this is the first study of the Ventana ALK (D5F3) CDx assay on ThinPrep slides.

The overall prevalence of ALK rearrangement with this method was 13.22% in our study, which is higher than the rates of 3% to 7% reported in previous studies [3–5]. We consider that the small sample size takes the major responsibility for this result. However, our results are in line with those of a previous study by our group showing that the prevalence of ALK rearrangement with FISH analysis on cytology specimens was 11.7% [21]. A higher prevalence of ALK rearrangement may be attributed to the presence of advanced disease stages among lung cancer patients who are diagnosed via cytology specimens.

The present study revealed a significant correlation between ALK ICC analysis on ThinPrep slides and ALK PCR/FISH analysis, especially when the semiquantified interpretation system was used. The sensitivity, specificity, PPV, and NPV of the ALK ICC test were 93.75%, 96.97%, 93.75%, and 96.97%, respectively, which are in line with a recent large-scale study of ALK IHC analysis [9]. In this study, 933 FFPE NSCLC tissue specimens were analyzed by both the ALK (D5F3) CDx assay and FISH. The results obtained with the ALK (D5F3) CDx assay were highly concordant with those obtained with the FISH assay, and the PPV and NPV of the ALK (D5F3) CDx assay were 86.0% and 96.3%, respectively.

The results of our study suggest that ALK ICC analysis on ThinPrep slides is a reliable ALK testing method that can be used for identification of patients suitable for treatment with crizotinib, particularly when tissue specimens are not available. An interesting finding was that the binary scoring algorithm recommended by manufacturer did not seem to be as valuable as previously reported [6, 9]. Four ALK-positive specimens that were missed when the binary scoring algorithm was used were positive for ALK rearrangement when the semiquantified interpretation system was used, and all 4 were also positive for ALK rearrangement when tested by PCR. The ICC staining pattern of these 4 cases showed intratumoral heterogeneity and moderate staining intensity, which is not a common phenomenon in ALK IHC based on FFPE tissue in that it often demonstrates diffuse strong tumor tissue staining [6]. As an extreme example, it showed moderate staining in 5% of tumor cells, and faint staining in the other 95% (Fig. 2d, e). However, we believe that the heterogeneity of ALK expression in ICC here is not a real heterogeneous expression. Because in the same sample of PCR detection, their results showed a clear positive, its CT value between 14-20, did not show heterogeneity. As mentioned above, this can mainly be attributed to a loss or decrease of immunogenicity caused by the fixation. For example, in the study of Sauter et al. [18], 13 of the 30 antibodies (43%) in a methanol-fixed cell block failed initial validation using IHC conditions that were established in the study laboratory for FFPE material, but when the IHC protocol was adjusted, immunogenicity was restored for most but not all of the antigens [18]. It is worth conducting a prospective, randomized study that explores the influence of alcohol or methanol fixation in ICC analysis. However,Based on the above data, the semiquantified interpretation system, due to its higher sensitivity, may be preferable for ALK ICC analysis on ThinPrep cytology slides, although there was no statistically significant difference between the 2 scoring systems.

There was one false-positive ALK ICC result in our study. This specimen showed faint staining in 50% of tumor cells and strong staining in another 5%, and was considered positive by both scoring systems (Fig. 2g, h) but negative on RT-PCR. It is well known that PCR analysis of ALK based on several common fusion points (Exon 2, 3, 6, 13, 14, 15, 17, 18 and 20) without including all the variants, leads to a negative result for ALK fusion protein expressed in ICC. Unfortunately, the ALK status of this patient did not have a chance to be confirmed by FISH analysis due to the lack of remaining specimens and non-receipt of treatment with crizotinib.

This is the first report of ALK ICC on ThinPrep slides. Further studies are required to corroborate the results obtained in this study. Furthermore, prospective studies that correlate a positive diagnostic test with patients’ response to treatment are required.

## Conclusion

ALK ICC analysis on ThinPrep slides is a reliable testing method for ALK rearrangement. It can be used as a stand-alone test to identify patients suitable for treatment with crizotinib when tissue specimens are not available for ALK analysis. With ALK ICC analysis on ThinPrep slides, the semiquantified interpretation system may be preferable to the binary scoring algorithm, as the test then has higher sensitivity without a reduction of specificity.

## Abbreviations

ALK: Anaplastic lymphoma kinase
FFEP: Formalin-fixed, paraffin-embedded
FISH: Fluorescence *in situ* hybridization
FNA: Fine-needle aspirate
ICC: Immunocytochemistry
IHC: Immunohistochemistry
NPV: Negative predictive value
NSCLC: Non–small-cell lung cancer
PPV: Positive predictive value
RT-PCR: Reverse-transcription polymerase chain reaction
